# Dose Effects of Histone Deacetylase Inhibitor Tacedinaline (CI-994) on Antipsychotic Haloperidol-Induced Motor and Memory Side Effects in Aged Mice

**DOI:** 10.3389/fnins.2021.674745

**Published:** 2021-10-06

**Authors:** Bryan McClarty, Guadalupe Rodriguez, Hongxin Dong

**Affiliations:** Department of Psychiatry and Behavioral Sciences, Feinberg School of Medicine, Northwestern University, Chicago, IL, United States

**Keywords:** aging, antipsychotics, histone deacetylases, histone acetylation, epigenetics

## Abstract

**Background:** Elderly patients treated with antipsychotic drugs often experience increased severity and frequency of side effects, yet the mechanisms are not well understood. Studies from our group indicate age-related histone modifications at drug targeted receptor gene promoters may contribute to the increased side effects, and histone deacetylase (HDAC) inhibitors entinostat (MS-275) and valproic acid (VPA) could reverse typical antipsychotic haloperidol (HAL) induced motor-side effects. However, whether such effects could be dose dependent and whether HDAC inhibitors could improve memory function in aged mice is unknown.

**Methods:** We co-treated selective class 1 HDAC inhibitor tacedinaline (CI-994) at different doses (10, 20, and 30 mg/kg) with HAL (0.05 mg/kg) in young (3 months) and aged (21 months) mice for 14 consecutive days, then motor and memory behavioral tests were conducted, followed by biochemical measurements.

**Results:** CI-994 at doses of 10 and 20 mg/kg could decrease HAL-induced cataleptic episodes but only 20 mg/kg was sufficient to improve motor coordination in aged mice. Additionally, CI-994 at 10 and 20 mg/kg mitigate HAL-induced memory impairment in aged mice. Biochemical analyses showed increased acetylation of histone marks H3K27ac and H3K18ac at the dopamine 2 receptor (D2R) gene (*Drd2*) promoter and increased expression of the *Drd2* mRNA and D2R protein in the striatum of aged mice after administration of CI-994 at 20 mg/kg.

**Conclusions:** Our results suggest CI-994 can reduce HAL-induced motor and memory side effects in aged mice. These effects may act through an increase of acetylation at the *Drd2* promoter, thereby restoring D2R expression and improving antipsychotic drug action.

## Introduction

Aged patients, especially those with psychosis and neurodegenerative disorders combining neuropsychiatric symptoms, are often prescribed antipsychotic and antidepressant medications to treat the severe symptoms (Cummings et al., 1994; Cohen-Mansfield, 2001; Aupperle, 2006; Kamble et al., 2008; Gareri et al., 2014). For instance, typical and atypical antipsychotics have been administered to help manage neuropsychiatric symptoms in Alzheimer’s disease (AD) patients, and studies have revealed modest efficacy of typical antipsychotics in dementia patients with neuropsychiatric symptoms (Schneider et al., 1990; Zaudig, 2000; Aupperle, 2006; De Deyn et al., 2013; Tampi et al., 2016). However, elderly (>65 yo) patients prescribed typical antipsychotics have shown an increase severity and frequency of extrapyramidal side effects (EPS), (Zaudig, 2000; Alexopoulos et al., 2004; Aupperle, 2006; Gareri et al., 2014). EPS typically include acute dyskinesias, tardive dyskinesia, and Parkinsonism’s (Blair and Dauner, 1992; Casey, 2004, 2006; Meltzer, 2013), all of which can be distressing and substantially affect the daily life and activities of these patients. Studies have indicated changes in the dopaminergic system of aged brains, which could be a potential factor influencing age-related sensitivities to typical antipsychotic drugs. Dopamine 2 receptors (D2Rs) are the main target for typical antipsychotics. An un-proportional ratio of dopamine and dopamine receptors, or excessive blockade of dopamine receptors in the basal ganglia circuits have been postulated to contribute to the pharmacodynamic changes in aged patients (Blair and Dauner, 1992), thus increasing the susceptibility of EPS in elderly patients to the antipsychotic drugs (Peluso et al., 2012; McClarty et al., 2018). Another study investigated the binding potential to D2Rs in the striatum of patients with neuropsychiatric symptoms of AD or AD alone and found that the binding potential in the patients with severe neuropsychiatric symptoms was significantly lower (Tanaka et al., 2003). These studies suggest that the dopaminergic system undergoes changes during aging, and such changes could influence drug efficacy and behavioral responses.

The mechanisms of aging-induced changes of the dopaminergic system in the brain remain unclear. Studies from our group have suggested that histone modifications at the D2R gene (*Drd2*) promoter, may be one of the factors contributing to the increased sensitivity of aged patients to antipsychotic side effects (Montalvo-Ortiz et al., 2017). We found that histone acetylation is decreased at the *Drd2* promoter, impacting the expression and function of D2R, thereby affecting drug action. HDAC inhibitors entinostat (MS-275) and valproic acid (VPA) were able to restore acetylation levels at *Drd2* and D2R expression, alleviating haloperidol (HAL) induced motor side effects (Montalvo-Ortiz et al., 2017). However, if such effects of HDAC inhibitors in the brain display a dose response has not been studied.

Histone modifications also play an important role in memory function during aging. Growing studies have used a wide range of HDAC inhibitors such as pan targeting HDACs or specific targeting HDAC for subclasses in aging and neurodegenerative models (Guan et al., 2009; Hahnen et al., 2008; Reolon et al., 2011). Studies have also shown that decreases or dysregulation of acetylation in the hippocampus greatly contributes to the increase of memory impairment in normal aging (Peleg et al., 2010; Castellano et al., 2012; Sen, 2015). For example, suberoylanilide hydroxamic acid (SAHA, a class 1 HDAC inhibitor) and VPA (broad acting) were able to ameliorate memory deficits, suggesting that HDAC inhibitors may have a potential to improve neuronal function and slow the progression or reverse cognitive deficits in aged patients (Reolon et al., 2011; Benito et al., 2015; Zhao et al., 2018).

Tacedinaline (CI-994), a novel selective class 1 HDAC inhibitor has been well investigated in cancer research, and recent evidence indicates that CI-994 has an impact on the central nervous system (Graff et al., 2014; Zhang et al., 2018; Fuller et al., 2019). In particular, studies have shown CI-994 could improve synaptic plasticity and memory function in mice (Graff et al., 2014; Fuller et al., 2019), however, there are no reports of whether CI-994 could reverse antipsychotic drug induced side effects in aged mice. In this study, we investigated the dose effects of CI-994 on typical antipsychotic drug HAL-induced motor and memory side effects in aged mice.

## Experimental Procedures

### Animals

Young (2–3 months old, total *n* = 36) and aged (21 months old, total *n* = 44) C57BL/6 male and female mice were equally distributed in each experimental group (*n* = 6–11 for behavioral tests, *n* = 4–6/group for biochemical analysis) from Charles River laboratories were used for this study. Animals were group housed on a 12-h light/dark cycle and given food and water ad libitum. All procedures in animals were performed according to NIH guidelines and the Current Guide for the Care and Use of Laboratory Animals (2011, eight edition) under a protocol approved by the Northwestern University Animal Care and Use Committee.

### Drugs

Antipsychotic drug HAL was purchased from Sigma (St. Louis, MO, United States), and CI-994 was purchased from MedChemExpress (Monmouth Junction, NJ, United States) with inhibition of IC_50_s of 0.9, 0.9, 1.2 μM for recombinant HDAC 1, 2 and 3. HAL (0.05 mg/kg) was first dissolved in 50 uL of glacial acetic acid and brought up to the final dose volume in 0.9% saline with pH adjusted with 0.1 M NaOH. We selected HAL at a dose of 0.05 mg/kg for our study because our previous work showed a range of 0.01–0.1 mg/kg of HAL to induce EPS-like behaviors in aged mice (Montalvo-Ortiz et al., 2017). For CI-994, 10, 20, or 30 mg/kg was dissolved in 2% DMSO and brought up the final dose volume in 0.9% saline with pH adjusted with 0.1 M NaOH. Drugs were prepared freshly on the day of administration. All compounds and vehicles were administered intraperitoneally (i.p.) at a constant volume of 10 μL/g of body weight once a day for 14 consecutive days (Supplementary Figure 1). CI-994 was injected 30 min before HAL administration. Immediately after drug administration, we carefully monitored animal physical condition and behaviors, including: respiratory stress, locomotor function and body weight. Respiratory distress was determined by breathing patterns in mice. If fast/short, labored breathing was observed for more than 30 min after injections, and this symptom persisted 1 day post injections was marked as respiratory distress. General locomotor activity was monitored by observation 5, 10, and 20 min after first day of injections. Then we measured locomotor activity 30 min after the first day of injections using the open field test. Locomotor function impairments were considered if a mouse movement was greatly reduced or no movement was shown compared to control mice for more than 30 min after drug administration. Body weight was measured daily, and any weight loss (>2 g) within 2–3 days was considered a significant side effect.

### Behavioral Tests

Mice were acclimated to a soundproof behavioral testing room 30 min prior to testing, and assays were performed during the light part of the 12-h light/dark cycle. Memory and motor function tests were conducted during the second week of drug administration (Supplementary Figure 1). The purpose of our experimental design was trying to reveal the maximum drug effects in 14 days including a long-term (14 days) and short-term (30–60 min) effects of HDAC inhibitor and antipsychotic drug on memory and motor behavior. Similar experimental designs have been reported in previous studies in our group or others (Dong et al., 2005; Martin et al., 2005; Xu et al., 2010; Song et al., 2016; Montalvo-Ortiz et al., 2017). The order of the behavioral test was as follow: Novel object recognition (NOR), rotarod, and catalepsy (Supplementary Figure 1). The behavioral tests and data analysis were conducted by one investigator that was blinded to age and treatment conditions.

#### Catalepsy

The Step-Down assay was used for cataleptic behavior. Briefly, a plastic rod (1-cm diameter) was suspended 3.5 cm above a laboratory bench in a soundproof behavioral room. Thirty minutes after drug injection, the front paws of the animal were placed on the rod while the hind paws rested on the bench. The duration of a cataleptic episode was defined as the time to step off from the rod during a 300-s trial (Fink-Jensen et al., 2011; Montalvo-Ortiz et al., 2017).

#### Motor Coordination

The TSE Rotarod System (Bad, Homburg, Germany) was used to assess motor coordination after drug treatment. Mice were placed on an accelerating rod (4–40 rpm during the first 5 min) for 10 min, and the latency to fall from the rod was recorded. A total of 3 trials were conducted with a 10-min inter-trial interval. The average latency to fall from the rod across the 3 trials was calculated and used for comparison (Kirschbaum et al., 2009; Montalvo-Ortiz et al., 2017).

#### Novel Object Recognition

Recognition memory was tested in an open plexiglass box (40 cm × 40 cm × 30 cm). Two sets of objects were used, and they were consistent in height and volume, but different in shape, color and texture. Mice were individually habituated to the test arena for 10 min on each of the 3 days prior to data acquisition. On the first day of habituation, a open field test for mouse locomotor activity and anxiety behavior were recorded to ensure the mice had no severe impairments in locomotor function and anxiety behavior due to antipsychotic drug and CI-994 administration. The experimental session consisted of 3 phases: acquisition trial (10 min), inter-trial interval (ITI; home-cage, 1 h), and retention trial (10 min). During acquisition, animals were recorded while exploring the arena with two glass, cylinder-shaped identical objects that are dark gray color (height of 8 cm and a diameter of 4 cm) placed diagonally across from each other. Following the ITI, during which one of the objects was replaced by a novel object that is plastic, prism-shaped with a yellow and blue color (6 cm length × 6.5 cm width × 6 cm height), in a counterbalanced manner, animals were placed back in the arena for the retention trial. The amount of time spent exploring each object during the acquisition and retention trial was scored by an experimenter blinded to the condition using two milliseconds stopwatches for precision. Exploration was defined as touching, leaning on the object or orienting the head towards the object and sniffing within <1.0 cm for at least 20 s to make sure the sensorial perception was not impaired. Climbing on top of the object was not counted as exploration. Between each trial, the arena and the objects were cleaned with 70% alcohol to eliminate olfactory traces. The time spent with each object during the retention trial was used to calculate the discrimination index (DI), which represents the difference in exploration time expressed as a proportion of the total time spent exploring the two objects. To calculate DI, the total time spent exploring the novel object was subtracted by the total time spent exploring the familiar object, divided by the total time spent exploring during the retention trial. The formula used to calculate DI is as follows:


$$=\frac{\begin{array}{c} \left(T\,o\,t\,a\,l\,t\,i\,m\,e\,s\,p\,e\,n\,t\,e\,x\,p\,l\,o\,r\,i\,n\,g\,n\,o\,v\,e\,l\,o\,b\,j\,e\,c\,t\right)\;\\ -\left(t\,o\,t\,a\,l\,t\,i\,m\,e\,s\,p\,e\,n\,t\,e\,x\,p\,l\,o\,r\,i\,n\,g\,f\,a\,m\,i\,l\,i\,a\,r\,o\,b\,j\,e\,c\,t\right)\; \end{array}}{T\,o\,t\,a\,l\,t\,i\,m\,e\,s\,p\,e\,n\,t\,e\,x\,p\,l\,o\,r\,i\,n\,g}$$

### Biochemical Analysis

For biochemical assessments, only 20 mg/kg CI-994 treatment groups were measured, as this dosage combined HAL was more effective on motor and memory function in aged mice.

#### Western Blot

After behavioral test, we collected brain tissues through a cardiac perfusion with 0.1M PBS solution for 1 min, to wash-out the blood from blood vessels in the mouse brain first. The brains were then removed and quickly dissected under ice with a dissecting scope. The striatum and prefrontal cortex were frozen at −80°C until ready for processing for molecular analysis. The abundance of dopamine 2 receptor (D2R) were determined in lysates of striatum and prefrontal cortex. Protein extraction was performed by homogenizing approximately 40 mg of tissue of each brain subregion in a mix of ice-cold RIPA buffer (catalog # R0278, Sigma-Aldrich, St. Louis, MO, United States) and protease inhibitor cocktail solution (catalog # PI78410, Fisher Scientific, Hampton, NH, United States). Tissues were processed first by using a cordless motor connected to a Teflon pestle (20 s; catalog # 12-141-362, Fisher Scientific, Hampton, NH, United States) followed by sonication with Branson 450 Digital Sonifier (amplitude 70%, 2–3 s; catalog # B450, Marshall Scientific, Hampton, NH). Samples were then centrifuged at 20,000 *g* for 10 min at 4°C and supernatants were collected for determination of total protein concentration. Protein content was measured using the Pierce^TM^ BCA protein assay kit (catalog # PIA53226, Fisher Scientific, Hampton, NH, United States, 2019). An equal amount of proteins (20 μg) were loaded and resolved through electrophoresis in 10% Criterion^TM^ TGX Stain-Free^TM^ Precast Gels at 100 V for 1.5 h (catalog # 5671035, Biorad, Hercules, CA, United States, 2019). Proteins were transferred onto a polyvinylidene difluoride membrane by using Trans-Blot^®^ Semi-Dry Electrophoretic Transfer Cell at 15 V for 1.5 h. Blots were exposed to 5% non-fat dry milk as blocking solution for 1 h at room temperature and immunostained overnight at 4°C with primary antibodies at 1:1000 dilution against D2R (Millipore, catalog # AB5084P, RRID#:AB_2094980, rabbit), and β-actin (Santa Cruz, catalog # sc-47778, mouse). The D2R antibody immunospecificity has been tested and cross-compared to other commercially available D2R primary antibodies and has been verified in brain lysates of D2R-KO mice (Stojanovic et al., 2017). The next day, membranes were incubated with 1:5000 dilution of HRP-conjugated secondary goat anti-mouse or anti-rabbit antibodies for 2 h at room temperature. Blotted proteins were detected and quantified using ChemiDoc^TM^MC imaging system (Biorad) and Image J Software. The levels of target protein expression were normalized to β-actin (Montalvo-Ortiz et al., 2017; Rodriguez et al., 2017).

#### Chromatin Immunoprecipitation Assay

The commercially available Magna ChIP^TM^G Tissue Kit (17-20000, Millipore) was used, and the published protocol was followed (Montalvo-Ortiz et al., 2014; Montalvo-Ortiz et al., 2017). Briefly, fragmented chromatin lysate was immunoprecipitated with 5 μg of antibody directed against H3K27ac and H3K18ac. The DNA-histone complex was incubated with Protein G Magnetic Beads overnight at 4°C. The DNA-histone complex was eluted from the beads and dissociated at 65 4°C for 2 h under high salt conditions. Proteins were digested using proteinase K treatment and the associated DNA was precipitated with 100% ethanol and resuspended in 50 μL of PCR grade water.

#### Quantitative Real-Time PCR

The levels of interaction between modified histones and the *Drd2* gene promoter were determined by measuring the amount of histone-associated DNA, isolated via chromatin immunoprecipitation (ChIP), using quantitative real-time PCR (qRT-PCR). The *Drd2* mRNA in the striatum and prefrontal cortex was isolated and extracted (Qiagen), then reverse transcription was performed on extracted mRNA (Quantabio) and quantitatively amplified using qRT-PCR. *Drd2* (forward: 5′- CTCTTTGGACTCAACAACA CAGA -3′, reverse: 5′- AAGGGCACGTAGAACGAGAC -3′) (Puighermanal et al., 2020) and β-actin (forward: 5′- TGTTACCAACTGGGACGACA-3′, reverse: 5′-ACCTGGG TCATCTTTTCACG-3′) primers were used. For ChIP, Drd2 promoter (forward: 5′- GCCCTATGGCTTGAAGGTAA -3′, reverse: 5′- GACAGGCGGCGCTAGAGT -3′) (Kurita et al., 2012) and β-actin promoter (forward: 5′-GAGACATTGAATGGGGCAGT-3′, reverse:5′-ATGAAGAGT TTTGGCGATGG-3′) were used. Input, immunoprecipitated DNA, and cDNA amplification reactions were run in triplicate in the presence of SYBR Green (Applied Biosystems) using QuantStudio 6 Flex Real Time PCR System (Applied Biosystems, Foster City, CA, United States). Ct values from each sample were obtained using the Sequence Detector 1.1 software. Ct values were normalized to endogenous gene, beta actin, to obtain a percent input. Fold differences (drug treated versus control) were then determined using the delta delta C_*t*_ method as a previous report (Livak and Schmittgen, 2001).

### Statistical Analysis

All statistical analyses were conducted using the GraphPad prism software (San Diego, CA, United States). Data are expressed as mean ± standard error of the mean (SEM). Two-way analysis of variance (ANOVA) was used to detect treatment effects followed by a multiple comparison’s analysis using Tukey’s post hoc method.

## Results

### Dose Dependent Response of CI-994 on Haloperidol Induced Motor Side Effects

For our study, we initially used a dose range of 10, 20, and 30 mg/kg for CI-994. This dose range was selected based on previous reports that have administered CI-994 in adult mice (Graff et al., 2014; Zhang et al., 2018; Zhao et al., 2018; Fuller et al., 2019). Given HDAC inhibitors and antipsychotic drugs have their own side effects, it was critical to find the optimal dose for aged mice. We found a dose range of 10–20 mg/kg for CI-994, which showed no visible signs of physiological and neurological side effects. However, administration of 30 mg/kg with haloperidol after 3 days induced a severe side effects including respiratory stress, motor function impairment and body weight loss, especially in aged mice, therefore, we excluded this dose in our experiments.

**FIGURE 1:**
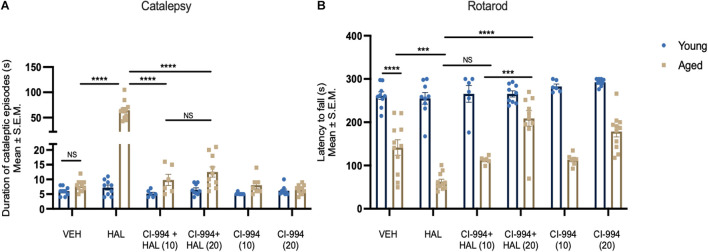
CI-994 mitigates HAL-induced motor side effects in aged mice. **(A)** Aged but not young mice increased cataleptic episodes after HAL administration. CI-994 at 10 and 20 mg/kg could efficiently blocked HAL-induced (10 mg/kg CI-994 + HAL vs HAL aged mice, *p* < 0.0001; 20 mg/kg CI-994 + HAL vs HAL aged mice, *p* < 0.0001) cataleptic behavior. However, there was no significant difference between 10 mg/kg CI-994 + HAL and 20 mg/kg CI-994 + HAL groups (*p* = 0.9997). CI-994 at 10 or 20 mg/kg alone do not affect cataleptic behavior **(B)**. Aged mice displayed an impairment of motor coordination tested by the Rotarod test (young VEH vs aged-VEH, *p* < 0.0001). HAL exacerbated a motor coordination deficit in aged mice (aged VEH vs aged HAL, *p* = 0.0001). CI-994 at 20 mg/kg but not 10 mg/kg significantly improve motor coordination in aged mice (10 mg/kg CI-994 + HAL vs HAL *p* = 0.2318; 20 mg/kg CI-994 + HAL vs HAL *p* < 0.0001). Additionally, a significant difference between 10 mg/kg CI-994 + HAL and 20 mg/kg CI-994 + HAL groups (*p* = 0.0001). Data represent mean ± SEM (*n* = 6–11/group). ***p < 0.001, ****p < 0.0001, NS, not significant.

We first tested cataleptic behavior after chronic administration of HAL, co-administration of HAL + CI-994, and CI-994 alone in young and aged mice. Two-way ANOVA revealed significant effects of age (*F*_1,92_ = 86.20, *p* < 0.0001) and drug (*F*_5,92_ = 61.82, *p* < 0.0001), and age × drug interaction (*F*_5,92_ = 56.13, *p* < 0.0001) on cataleptic episodes (Figure 1A). Post-hoc analysis revealed aged mice administered HAL displayed severe cataleptic behavior compared to aged VEH mice (*p* < 0.0001). However, CI-994 at 10 and 20 mg/kg doses significantly decreased HAL induced cataleptic behavior (both *p* < 0.0001), and no significant difference between 10 and 20 mg/kg groups. Also, no significant differences were found in young mice between treatment groups. These results suggest that chronic HAL administration induces severe cataleptic behavior in aged mice, and CI-994 at 10–20 mg/kg are able to mitigate this side effect.

Next, we evaluated the impact of CI-994 on HAL-induced motor coordination deficits in young and aged mice using the rotarod test. Two-way ANOVA revealed significant effects of age (*F*_1,92_ = 336.0, *p* < 0.0001) and drug (*F*_5,92_ = 8.056, *p* < 0.0001), and an age x drug interaction (*F*_5,92_ = 7.537, *p* < 0.0001) on the latency to fall from the rod (Figure 1B). Post-hoc analysis showed a significant decline of motor coordination in aged mice compared to young mice (*p* < 0.0001) and HAL accelerated a decline of motor coordination in aged mice (*p* = 0.0001). When CI-994 was administered with HAL, only 20 mg/kg showed a significant improvement in motor coordination compared HAL (*p* < 0.0001) alone. Additionally, aged mice with 20 mg/kg CI-994 + HAL showed a significant increase in the latency to fall compared to 10 mg/kg CI-994 + HAL (*p* = 0.0048). No statistical differences were found in young mice between treatment groups. These results suggest that motor coordination declines in normal aging, and chronic administration of HAL exacerbates the motor coordination impairment. However, CI-994 at a dosage of 20 mg/kg, is sufficient to mitigate this side effect and improve motor coordination in aged mice.

### Dose Dependent Effects of CI-994 on Haloperidol Induced Memory Impairment

**FIGURE 2:**
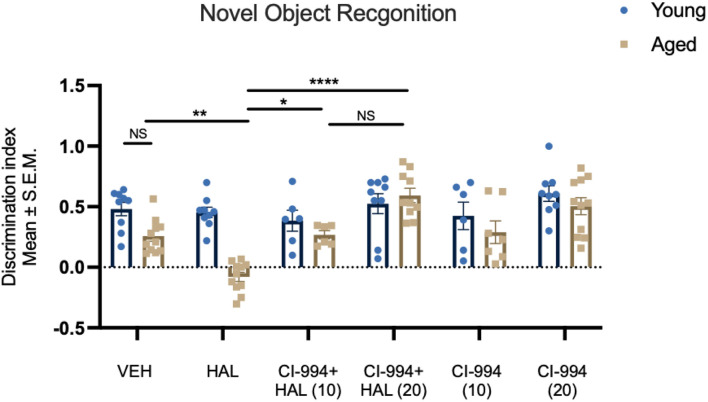
CI-994 restores recognition memory impaired by HAL in aged mice. Aged mice did not display a decrease in recognition memory tested by NOR (VEH-aged vs VEH-young, *p* = 0.2752), however, HAL induced a memory impairment (aged HAL vs aged VEH, *p* = 0.0042). CI-994 at 10 and 20 mg/kg improved the memory function in a dose dependent manner (aged CI-994 10mg/kg + HAL vs aged HAL, *p* = 0.0249; aged CI-994 20 mg/kg + HAL vs. aged HAL, *p* < 0.0001). No significant difference between 10 mg/kg CI-994 + HAL and 20 mg/kg CI-994 + HAL groups (*p* = 0.0576). Additionally, there was no difference between CI-994 alone and VEH groups in aged mice (aged CI-994 10 mg/kg vs. aged VEH, *p* > 0.9999; aged CI-994 20 mg/kg vs. aged VEH, *p* = 0.1109). Data represent mean ± SEM (*n* = 6–11/per group). *p < 0.05, **p < 0.01, ****p < 0.0001, NS, not significant.

In addition to motor effects, in this study, we also investigated whether HAL could induce a memory impairment in aged mice and whether HDAC inhibition could reverse such an effect. We measured recognition memory through the novel object recognition (NOR) test. Two-way ANOVA revealed effects of age (*F*_1,92_ = 20.83, *p* < 0.0001) and drug (*F*_5,92_ = 10.87, *p* < 0.0001), and an age × drug interaction (*F*_5,92_ = 5.298, *p* = 0.0003) in the recognition memory after HAL and CI-994 administration (Figure 2). Post-hoc analysis revealed no significant difference between young and aged VEH mice in the discrimination index (DI). However, there was a significant decrease of DI in HAL-treated aged mice as compared to VEH-treated aged control (*p* = 0.0042). Administration of CI-994 with HAL showed a significant increase of DI at a dosage of 10 mg/kg (*p* = 0.0249) and 20 mg/kg (*p* < 0.0001) as compared to HAL treated alone in aged mice. We found a trend of increased memory function in aged mice after 20 mg/kg CI-994 + HAL compared to 10 mg/kg CI-994 + HAL, although the statistical analysis did not reach the significant (*p* = 0.0576). Also, no significant differences were found in young mice between treatments groups. This data suggests that chronic administration of HAL induces recognition memory impairment and CI-994 at 10 and 20 mg/kg could restore memory function impaired by HAL in aged mice, and CI-994 + HAL at a dosage of 20 mg/kg tends to be more efficient.

### Effect of CI-994 on *Drd2* Gene Expression in the Prefrontal Cortex and Striatum of Aged Mice

**FIGURE 3:**
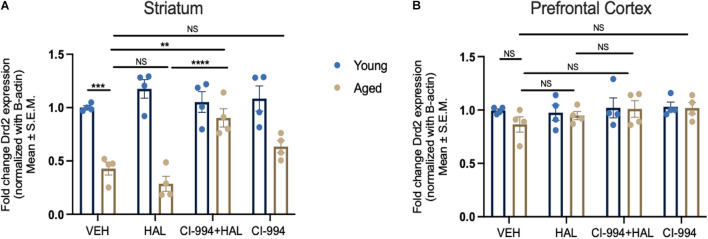
CI-994 restores *Drd2* gene expression in the striatum but not in the prefrontal cortex of aged mice. **(A)** Aged mice showed a significant decrease of *Drd2* mRNA expression as compared to young mice (aged VEH vs. young VEH, *p* = 0.0007), however, HAL treatment did not impact *Drd2* mRNA expression (aged VEH vs. aged HAL, *p* = 0.9050). CI-994 20 mg/kg with HAL significantly increased *Drd2* expression (aged VEH vs. aged CI-994 + HAL 20 mg/kg, *p* = 0.0060; aged HAL vs. aged CI-994 + HAL 20 mg/kg, *p* < 0.0001). However, CI-994 20 mg/kg treatment alone did not have an effect on *Drd2* mRNA expression (aged CI-994 20 mg/kg vs. aged VEH, *p* = 0.6034). **(B)** Chronic treatment of CI-994 at 20 mg/kg alone or combined with HAL did not increase *Drd2* mRNA expression in the prefrontal cortex of aged mice. Data represent mean ± SEM (*n* = 4/per group). **p < 0.01, ***p < 0.001, ****p < 0.0001, NS, not significant.

We determined whether CI-994 could mediate *Drd2* gene expression in the prefrontal cortex and striatum, which was conducted through qRT-PCR. Two-way ANOVA revealed significant effects of age (*F*_1,24_ = 84.16, *p* < 0.0001), drug (*F*_3,24_ = 4.822, *p* = 0.0091), and an age × drug interaction (*F*_3,24_ = 7.427, *p* = 0.0011) of *Drd2* mRNA expression in the striatum (Figure 3A). Post hoc analysis showed a significant decrease in *Drd2* mRNA expression in aged mice as compared to young mice (*p* = 0.0007) in VEH groups. However, a significant increase of *Drd2* mRNA expression in the striatum of CI-994 + HAL group as compared to the groups of VEH (*p* = 0.0060) and HAL alone (*p* < 0.0001) in aged mice. No significant differences were found in young mice between treatment groups. Additionally, *Drd2* gene expression was not statistically different between young and aged mice in VEH groups and across treatment in the prefrontal cortex (Figure 3B).

### Effect of CI-994 on D2R Protein Expression in the Prefrontal Cortex and Striatum of Aged Mice

**FIGURE 4:**
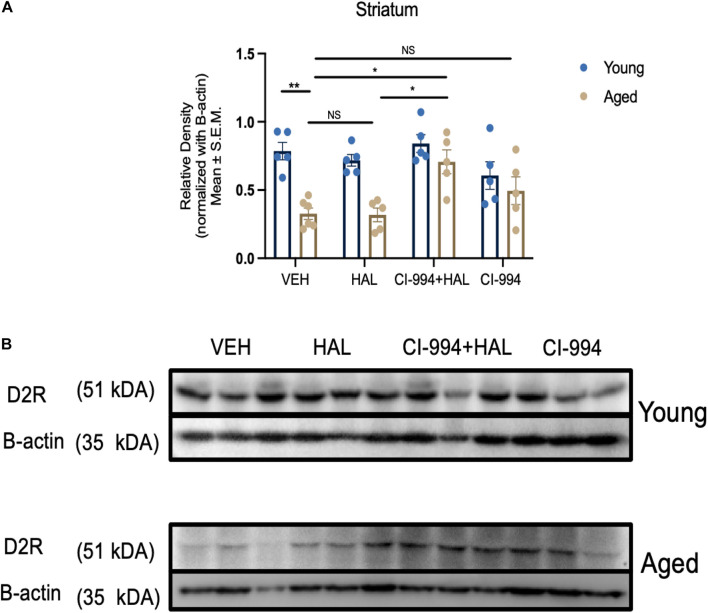
CI-994 restores D2R protein expression in the striatum of aged mice. **(A)** Quantitative analysis of D2R protein expression levels in the young and aged striatum through immunoblotting. Aged mice showed a significant decrease in D2R expression (aged VEH vs. young VEH, *p* = 0.0011), however, HAL treatment during aging did not impact D2R expression (aged VEH vs. aged HAL, *p* > 0.9999). CI-994 + HAL 20 mg/kg treatment significantly increased D2R expression (aged VEH vs. aged CI-994 + HAL 20 mg/kg, *p* = 0.0102; aged HAL vs. aged CI-994 + HAL 20 mg/kg, *p* = 0.0130). However, CI-994 20 mg/kg treatment did not have an effect on D2R expression (aged CI-994 20 mg/kg vs. aged VEH, *p* = 0.6779). **(B)** Western blot images from panel **(A)**. Each band represents a different biological sample in each treatment group. Data represent mean ± SEM (*n* = 5–6/per group). *p < 0.05, **p < 0.01, NS, not significant.

**FIGURE 5:**
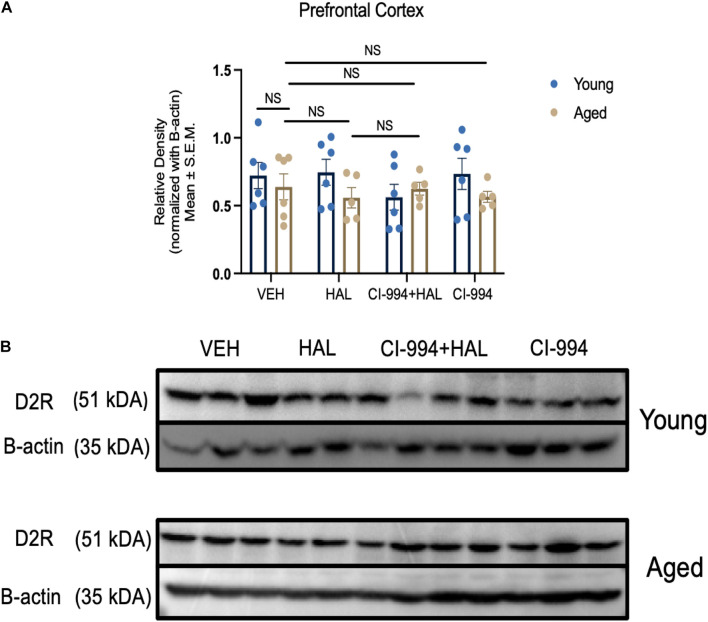
CI-994 does not affect D2R protein expression in prefrontal cortex of aged mice **(A)** Quantitative analysis of D2R protein expression levels in the young and aged striatum through immunoblotting. Chronic treatment of CI-994 at 20 mg/kg in combination with HAL did not increase D2R protein expression in the prefrontal cortex of aged mice. **(B)** Western blot images from panel **(A)**. Each band represents a different biological sample in each treatment group. Data represent mean ± SEM (*n* = 5–6/per group).

Our previous study indicates that D2R protein expression decreased in the striatum in aged mice and HDAC inhibitors VPA and MS-275 could modulate D2R expression (Montalvo-Ortiz et al., 2017). In this study, we determined whether CI-994 also could mediate D2R expression in the prefrontal cortex and striatum. Two-way ANOVA revealed significant effects of age (*F*_1,33_ = 29.56, *p* < 0.0001), drug (*F*_3,33_ = 5.233, *p* = 0.00046), and an age × drug interaction (*F*_3,33_ = 3.130, *p* = 0.0387) in D2R expression in the striatum (Figures 4A,B). Post hoc analysis showed a significant decrease in D2R expression in aged mice as compared to young mice (*p* = 0.0011) in VEH groups. Administration of CI-994 with HAL showed a significant increase of D2R expression in the striatum as compared to VEH (*p* = 0.0102) and HAL alone (*p* = 0.0130) in aged mice. No significant differences were found in young mice between treatment groups. However, D2R expression was not statistically different between young and aged mice in VEH groups and across treatment in the prefrontal cortex (Figures 5A,B). These results indicate that chronic administration of CI-994 at dose 20 mg/kg could rescue D2R expression in the striatum, but not prefrontal cortex, suggesting the effect of CI-994 on D2R expression is regional specific.

### CI-994 Impact on Histone Modifications in the Prefrontal Cortex and Striatum of Aged Mice

**FIGURE 6:**
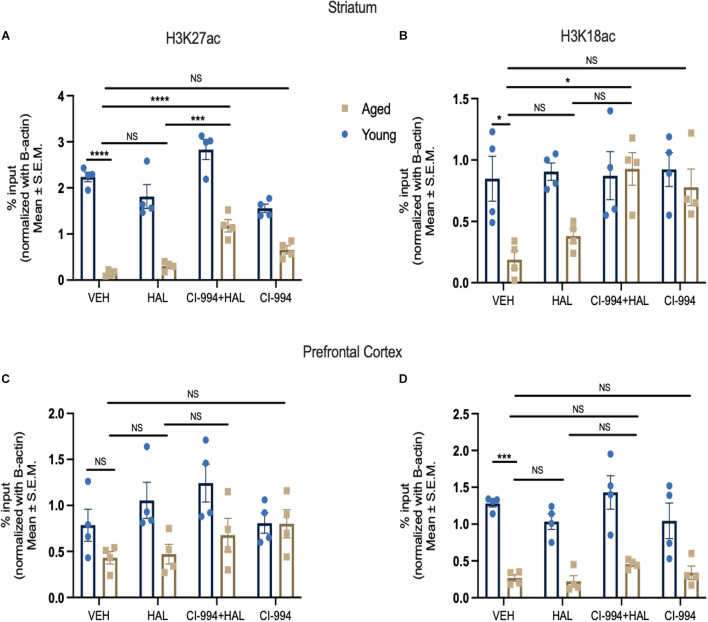
CI-994 regulates histone acetylation at *Drd2* promoter in the stratum but not prefrontal cortex. **(A,B)** Histone acetylation H3K27ac and H3K18ac levels at *Drd2* promoter in the striatum measured as % input. **(C,D)** Histone acetylation H3K27ac and H3K18ac levels at Drd2 promoter in the prefrontal cortex. Aged mice showed a significant decrease of H3K27ac at the *Drd2* promoter in the striatum (aged VEH vs. young VEH, *p* < 0.0001) and a decrease of H3K18ac at the *Drd2* promoter (aged VEH vs. young VEH, *p* = 0.0357). HAL treatment did not change H3K27ac level at *Drd2* (aged VEH vs. aged HAL, *p* = 0.2239) and H3K18ac (aged VEH vs. aged HAL, *p* = 0.9682) in aged mice. Co-treatment of CI-994 (20 mg/kg) and HAL significantly increased H3K27ac level at *Drd2* (aged VEH vs. aged CI-994 + HAL, *p* = 0.0006; aged HAL vs. CI-994 + HAL, *p* = 0.00043) and H3K18ac (aged VEH vs. CI-994 + HAL, *p* = 0.0357) in the striatum. CI-994 20 mg/kg alone did not have a significant impact on H3K27ac (aged CI-994 + HAL 20 mg/kg vs. aged VEH, *p* = 0.2239), however, a trend of increased H3K18ac (aged CI-994 + HAL 20 mg/kg vs. aged VEH, *p* = 0.0785) levels at *Drd2* in the striatum was found, but this did not reach statistical significance. In the prefrontal cortex, a significant decrease of H3K18ac but not H3K27 was found (aged VEH vs. young VEH, *p* < 0.0004) in the prefrontal cortex. However, there was no significant differences of H3K27ac or H3K18ac levels were found between treatment groups. Data represent mean ± SEM (*n* = 4/per group). *p < 0.05, ***p < 0.001, ****p < 0.0001, NS, not significant.

In order to further confirm epigenetic alterations in aging contributed to increased side effects of antipsychotic drugs, we investigated the status of histone acetylation at typical antipsychotic drug target, *Drd2* promoter, and determined if chronic administration of CI-994 can alter the levels of histone acetylation at the *Drd2* promoter in aged mice. We selected histone 3 lysine 27 (H3K27) and 18 acetylation (H3K18) as these histone marks have been associated with transcriptional regulation (Pham et al., 2012; Chen et al., 2019). Two-way ANOVA revealed significant effects of age (*F*_1,24_ = 235.8, *p* < 0.0001) and drug (*F*_3,24_ = 19.91, *p* < 0.0001), and an age x drug interaction (*F*_3,24_ = 6.034, *p* = 0.0033) in H3K27ac levels binding at the *Drd2* promoter in the striatum (Figure 6A). The post-hoc analysis revealed a significant decrease of H3K27ac levels in aged mice as compared to young mice in VEH groups (*p* < 0.0001). CI-994 at 20 mg/kg showed a significant increase of H3K27ac levels binding to the *Drd2* promoter as compared to VEH (*p* = 0.0006), and HAL (*p* = 0.0043) treated alone in aged mice. No significant differences were found in young mice between treatment groups. Similarly, two-way ANOVA revealed significant effects of age (*F*_1,24_ = 11.21, *p* = 0.0027), drug (*F*_3,24_ = 3.532, *p* = 0.0299), and an age x drug interaction (*F*_3,24_ = 3.022, *p* = 0.0493) in H3K18ac levels at the *Drd2* promoter in the striatum (Figure 6B). Post-hoc analysis revealed a significant decrease of H3K18ac binding at the *Drd2* promoter in aged as compared to young mice in VEH groups (*p* = 0.00357). However, CI-994 at dose 20 mg/kg showed an increase of H3K18ac levels binding to the *Drd2* promoter of as compared to VEH treated group (*p* = 0.00137) in aged mice. No significant differences were found in young mice between treatment groups.

We next evaluated the epigenetic changes occurring at *Drd2* promoter in the prefrontal cortex. Two-way ANOVA revealed only a significant effect of age (*F*_1,24_ = 11.72, *p* = 0.0022) in H3K27ac levels (Figure 6C), and effect of age (*F*_1,24_ = 87.47, *p* < 0.0001) in H3K18ac levels binding to the *Drd2* promoter (Figure 6D) in the prefrontal cortex. Post-hoc analysis revealed a significant decrease in H3K18ac levels binding at the *Drd2* promoter, but not H3K27ac levels, in aged mice as compared to young mice in VEH groups (*p* = 0.0004). No significant differences were found in both young and aged mice between treatment groups. Our results indicate that during normal aging, there is a decrease in H3K27ac and H3K18ac levels at the *Drd2* promoter in the striatum and a decrease of H3K18ac levels at the *Drd2* promoter in prefrontal cortex. In addition, CI-994 could rescue histone acetylation in aged mice, specifically in the striatum, which may modulate the antipsychotic induced side effects in aged mice through regulation of D2R expression in response to antipsychotic actions.

## Discussion

In this study, we demonstrated that a selective class 1 HDAC inhibitor, CI-994, can reduce the severity of motor and memory side effects in HAL treated aged mice. In particular, CI-994 at doses of 10–20 mg/kg were effectively to alleviate HAL-induced cataleptic behavior, however, only CI-994 at 20 mg/kg significantly improved motor coordination in aged mice. Additionally, CI-994 at both 10–20 mg/kg mitigate HAL induced memory impairment, and it seems that CI-994 at dose of 20 mg/kg was more efficient for improving recognition memory function. The effect of CI-994 on motor function may be acting through the regulation of histone modifications at the *Drd2* promoter, thus mediating D2R expression in the striatum. To our knowledge, we are the first to report the benefits of CI-994 in prevention of antipsychotic drug induced motor and memory side effects in aged mice.

In this study, we not only investigated a new HDAC inhibitor, CI-994, but also tested the dose response effects of this inhibitor on antipsychotic drug induced motor and memory side effects. We selected 3 doses for CI-994, ranging from 10 to 30 mg/kg, and found chronic administration of CI-994 at 30 mg/kg alone or combined with HAL induced severe side effects, including: increased respiratory distress, slower/no movement and weight loss especially in aged mice, however, mice administered CI-994 at doses of 10–20 mg/kg responded well and did not display such side effects. These results indicate that HDAC inhibitor itself could induce some side effects, or even results in toxic outcomes, which could be an impendence in preclinical research and clinical application (Subramanian et al., 2010; Gao et al., 2019; Shah, 2019). Therefore, when testing a new compound in animal models, it is critical to carefully evaluate dose responses by measuring the physiological outcomes, in order to identify the optimal dose range. Based on our screen, we selected doses of 10–20 mg/kg to continue our experiments and conduct the behavioral and memory tests. We found that CI-994 at 10 mg/kg was only effective in decreasing cataleptic behavior, in contrast, CI-994 at 20 mg/kg was effective in decreasing cataleptic episodes and improving motor coordination (Figure 1). Although other motor assessments such as hind limb grasping and gait analysis could be applied to measure motor behavior, our results indicate both catalepsy and rotarod tests are sensitive and stable for motor function evaluation in aged mice. In fact, catalepsy has been widely used for testing extrapyramidal side effects induced by antipsychotics in preclinical studies (Bardin et al., 2006; Fink-Jensen et al., 2011; Nishchal et al., 2014; Luciani et al., 2020).

In our study, we also confirmed our previous reports that D2R expression was decreased in the striatum of aged mice (Montalvo-Ortiz et al., 2017) and demonstrated another HDAC inhibitor CI-994 could rescue D2R expression in the aged striatum in addition to VPA and MS-275 (Montalvo-Ortiz et al., 2017). This effect was due to HDAC inhibitor mediated histone acetylation at the *Drd2* gene promoter, as we found *Drd2* mRNA expression was correlated with these changes in the striatum of aged mice. This prediction was supported by previous works, as various human and animal studies have shown significant decreases of D2R expression in the basal ganglia during aging, which has substantial impacts on target receptor binding capacities (Antonini et al., 1993; Rinne et al., 1993; Volkow et al., 1996; Tanaka et al., 2003; Hoekzema et al., 2010; Shiroma et al., 2010). Various factors such as sleep deprivation and abnormal dopaminergic neurotransmission, and post-transcriptional mechanisms could affect D2R expression during aging (Sakata et al., 1992; Meng et al., 1999; Ishibashi et al., 2009; Volkow et al., 2012). We proposed that histone modifications at the *Drd2* promoter is a contributing factor for D2R response to antipsychotics. Our reports demonstrated that changes of histone acetylation and methylation occurred at the *Drd2* promoter region in the striatum in aged mice and HDAC inhibitors VPA and MS-275 can modify histone acetylation at *Drd2* promoter by increasing acetylation (H3K27ac, H3K9ac, H4K12ac) levels and restore D2R expression (Montalvo-Ortiz et al., 2017). In this study, we further confirmed H3K27ac decreased with age, however, we also found another histone acetylation mark H3K18ac was decreased and CI-994 rescued acetylation of H3K27ac and H3K18ac at *Drd2* promoter in the striatum of aged mice. This new information provides additional evidence that histone modifications play a critical role in drug efficacy and HDAC inhibition in conjunction with antipsychotic drug treatment, which can improve motor side effects through modulation of histone acetylation at the *Drd2* promoter, subsequently modulating D2R expression and function (Montalvo-Ortiz et al., 2017). This statement is supported by our previous study, as we found the changes of downstream of D2R signaling in our previous study (Montalvo-Ortiz et al., 2017) as well as our current behavioral outcomes, which reflect the function has been changed in D2Rs. Therefore, our study further supports the beneficial effects of HDAC inhibitors on antipsychotic action in aged mice.

Typical antipsychotic drugs not only increase the risk of EPS, but also increase the risk of cognitive impairment (Jeste et al., 1999; Dolder and Jeste, 2003). Many studies have reported impaired memory function after given HAL (Terry et al., 2002; Hou et al., 2006; Abdel-Salam et al., 2012; Ozdemir et al., 2012; Ning et al., 2017), and our results are consistent with these findings. The mechanisms in which HAL induces memory impairment is not clear. We found that CI-994 was able to reverse HAL induced memory impairment suggesting that histone modifications were involved. In fact, previous studies have found that CI-994 was able to improve memory function in both aging and AD mice through increasing histone acetylation (Graff et al., 2014; Fuller et al., 2019). The prefrontal cortex is one of various brain areas that is involved in several memory processes. However, we did not find significant changes of D2R expression across aging and treatment in the prefrontal cortex. Additionally, CI-994 did not affect the selected histone acetylation marks at the *Drd2* promoter in the prefrontal cortex. A possible explanation could be that D2R in the prefrontal cortex is not involved in recognition memory processes and CI-994 improving memory function in HAL treated aged mice may be through regulating histone acetylation at other genes that are associated with memory function or indirectly improved memory by decreasing drug induced side effects. Various studies have shown that histone modifications change at memory-related genes contribute to age and disease-related cognitive decline, and HDAC inhibitors can reverse such effects (Francis et al., 2009; Kilgore et al., 2010; Peleg et al., 2010; Graff et al., 2014; Yao et al., 2014; Benito et al., 2015; Cooper et al., 2020). Therefore, in future studies we plan to investigate whether HAL administration could affect the histone acetylation changes occurring at memory and synaptic plasticity-related genes in aged mice, then to determine whether HDAC inhibitors could mediate such effects. This work would help reveal the epigenetic mechanisms of how CI-994 in conjunction with HAL improve memory function through histone acetylation in memory-related transcriptional programs impacted by HAL.

We selected the striatum and the PFC to study the molecular mechanisms of antipsychotic induced severe side effects in aged mice due to their anatomical and functional relevant to neuropsychiatric disorders and are areas enriched in D2R expression. We expect that D2R expression and function change during aging, which could influence antipsychotic drug actions, and these changes may be regulated by epigenetic mechanisms. However, in our study, we only found D2R expression decreased in the striatum, but not in the PFC. Additionally, CI-994 does not have an effect on D2R expression in PFC in aged mice, suggesting the striatum is more critical in response to the antipsychotic drug induced side effects in aged mice. This prediction was supported by previous works, as various human and animal studies have shown significant decreases of D2R expression in the basal ganglia, but not in the cortex during aging, which has substantial impacts on target receptor binding capacities (Antonini et al., 1993; Rinne et al., 1993; Volkow et al., 1996; Tanaka et al., 2003; Hoekzema et al., 2010; Shiroma et al., 2010). Our study also indicates the epigenetic regulation in the brain are gene and regional specific. Therefore, future studies for other brain regions and different receptor genes targeted by antipsychotic drugs are needed.

Histone acetylation is one of the most common histone modifications that significantly modulates the gene transcription and functional consequences (Forsberg and Bresnick, 2001; Gregory et al., 2001; Verdone et al., 2005; Verdone et al., 2006). However, other epigenetic mechanisms such as histone and DNA methylation also could affect the antipsychotic drug efficacy and memory function (Penner et al., 2011; Guidotti and Grayson, 2014; Sen, 2015; Snigdha et al., 2016; Seo et al., 2018). Additionally, besides HDACs, histone acetyltransferases (HATs) also play an important role during the balance of the status of histone acetylation and deacetylation and play a critical role in neuronal function, which may be influencing D2R response to antipsychotic drug as well. Future studies are still necessary to investigate other epigenetic mechanisms occurring at antipsychotic drug target receptor gene promoters, to further prove that histone modifications play a significant role in the aging brain, which influences drug efficacy.

In summary, our results from this study demonstrate that selective class 1 HDAC inhibitor, CI-994, could reduce the age-related sensitivity to typical antipsychotic drug HAL-induced motor side effects. This effect is likely that CI-994 improved the expression and functionality of D2R through modulation of histone acetylation at the *Drd2* promoter, subsequently affecting the gene expression. CI-994 also restored memory function that was impaired due to HAL administration in aged mice. Our study suggests that CI-994 may have the potential to prevent antipsychotic drug induced motor and memory side effects in the elderly. This work provides additional information to support that HDAC inhibitors could serve as a promising adjunct therapy in conjunction with typical antipsychotic drug HAL in the elderly population.

## Data Availability Statement

The raw data supporting the conclusions of this article will be made available by the authors, without undue reservation.

## Ethics Statement

The animal study was reviewed and approved by Institutional Animal Care and Use Committee (IACUC) at Northwestern University.

## Author Contributions

BM and HD designed the experiments and supervised the project. BM was responsible for mice handling, drug preparations, i.p. injections, conducted all behavior experiments and data collection, carried out western blotting, carried out all data analysis and presentation, and wrote the manuscript. GR assisted in drug preparations. BM and GR collected mouse brain tissue, carried out chIP+qPCR. HD and GR revised the manuscript. All authors read and approved the final version of the manuscript.

## Conflict of Interest

The authors declare that the research was conducted in the absence of any commercial or financial relationships that could be construed as a potential conflict of interest.

## Publisher’s Note

All claims expressed in this article are solely those of the authors and do not necessarily represent those of their affiliated organizations, or those of the publisher, the editors and the reviewers. Any product that may be evaluated in this article, or claim that may be made by its manufacturer, is not guaranteed or endorsed by the publisher.

## Supplementary Material

Supplementary Figure 1 Schematic of Experimental Procedure. Young and aged mice were treated with VEH, HAL, CI-994 + HAL, or CI-994 alone for 14 days. Behavioral tests were completed during days 8–13 with order: Novel object recognition (NOR, day 9–11), rotarod (day 12), and catalepsy (day 13). On day 14, the striatum and prefrontal cortex were dissected and collected for further biochemical assessments.
