# Supplementary material for: Dose Effects of Histone Deacetylase Inhibitor Tacedinaline (CI-994) on Antipsychotic Haloperidol-Induced Motor and Memory Side Effects in Aged Mice
Source: Front Neurosci. 2021 Oct 6;15:674745. doi: 10.3389/fnins.2021.674745 (PMC8526546; doi:10.3389/fnins.2021.674745)
Supplement: Supplementary Figure 1 — Schematic of Experimental Procedure. Young and aged mice were treated with VEH, HAL, CI-994 + HAL, or CI-994 alone for 14 days. Behavioral tests were completed during days 8–13 with order: Novel object recognition (NOR, day 9–11), rotarod (day 12), and catalepsy (day 13). On day 14, the striatum and prefrontal cortex were dissected and collected for further biochemical assessments. [file Presentation_1.ppt]

## Slide 1
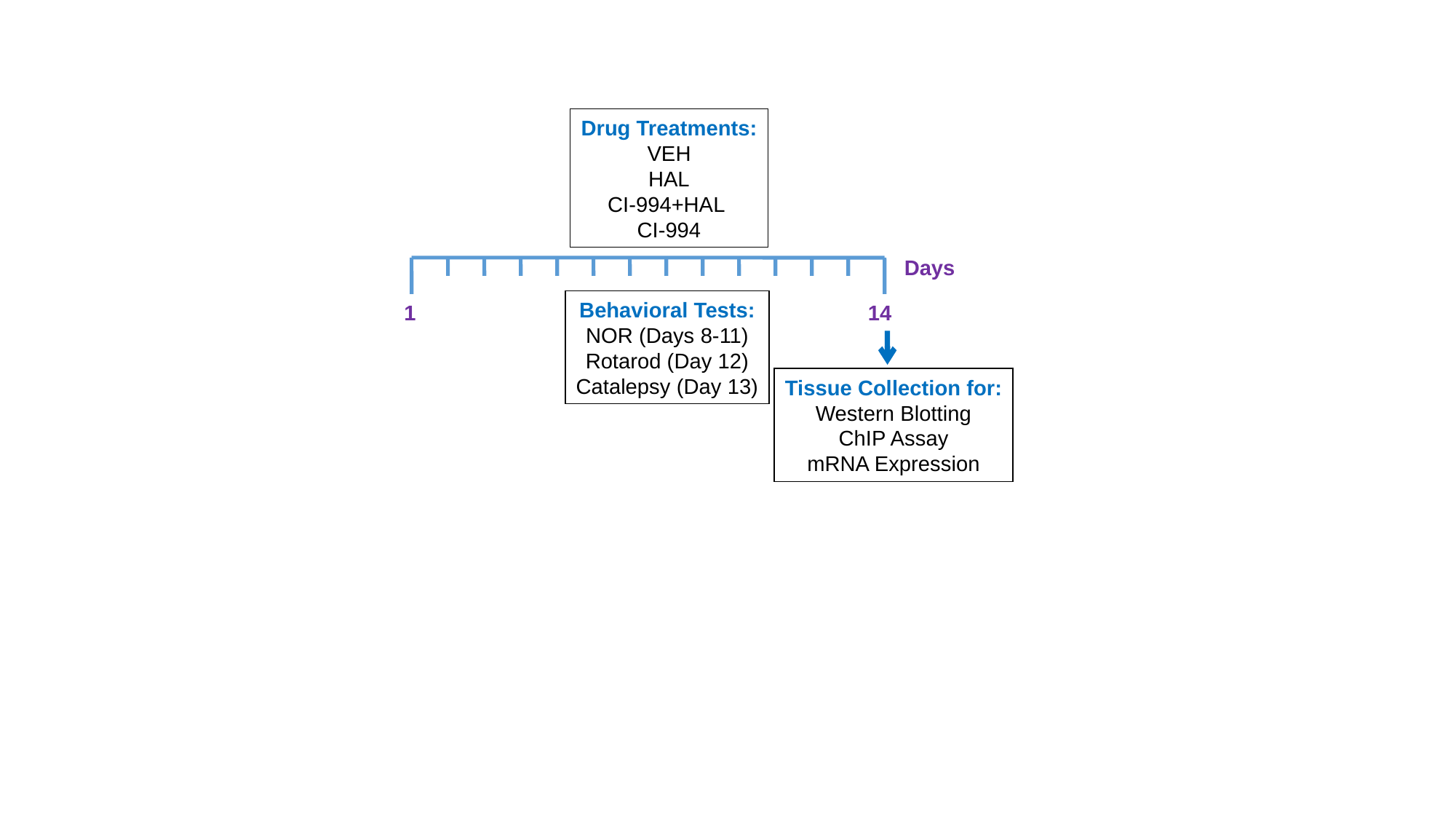

Drug Treatments:
VEH
HAL
CI-994+HAL
CI-994
Days
1
14
Behavioral Tests:
NOR (Days 8-11)
Rotarod (Day 12)
Catalepsy (Day 13)
Tissue Collection for:
Western Blotting
ChIP Assay
mRNA Expression
